# INTEREST GROUP SESSION—ABUSE, NEGLECT AND EXPLOITATION OF ELDERLY PEOPLE: DAILY REFLECTIONS OF DEMENTIA CAREGIVERS: MICRO-LONGITUDINAL APPROACHES TO UNDERSTANDING ELDER ABUSE AND NEGLECT

**DOI:** 10.1093/geroni/igz038.2001

**Published:** 2019-11-08

**Authors:** Carolyn E Pickering, Zach Gassoumis

**Affiliations:** 1 University of Texas Health Science Center San Antonio, San Antonio, Texas, United States; 2 Leonard Davis School of Gerontology, University of Southern California, Los Angeles, California, United States

## Abstract

Studies with clinic samples have found approximately 50% of family caregivers self-report engaging in abusive and neglectful acts towards the person with dementia whom they assist. Despite this, interventions to reduce and prevent elder abuse and neglect in dementia caregiving are lacking. To develop targeted interventions, the field still has much to learn about what happens during single incidents of elder abuse and neglect including (1) the types of tactics used (2) contextually-based risk/protective factors and (3) circumstances surrounding acts. This symposium will advance discussions on these topics through presentation of pilot data from a micro-longitudinal study on abuse and neglect within dementia family caregiving. Micro-longitudinal methods, such as daily diary studies, rely on intensive longitudinal measures over shorter periods of time. This approach can better ascertain ecologically-valid factors and identify temporal patterning between variables than traditional longitudinal and cross-sectional methods. First, we will provide an overview of the pilot project with family caregivers (N=50) completing diaries for 21 days. The second presenter will discuss data on the co-occurrence of different types of elder abuse and neglect as they manifest in daily lives of family caregivers, and lead discussion on measurement considerations. The next presenter will discuss data on contextually-based risk and protective factors for abuse and neglect that occur during daily caregiving activities. The final presentation will discuss findings on caregivers’ perceptions of the circumstances surrounding abusive and neglectful behaviors. Discussion will focus on how these findings, and methods, can be used to advance intervention development for the field.

